# Charleston Preparatory Medical School

**Published:** 1852-03

**Authors:** 


### THE MEDICAL EXAMINER.

### PHILADELPHIA, MARCH, 1852.

We beg leave to call the attention of our readers to the following excellent system of summer instruction, the advantage of similar schools having been amply tested by students who remain in Philadelphia during the summer months. There are two in successful operation here, viz.: the Philadelphia Association for Medical Instruction, whose announcement will be found in our advertising sheet, and the Philadelphia Medical Institute.

### CHARLESTON PREPARATORY MEDICAL SCHOOL.

Preparatory Schools of Medicine have, for a long time, been very much needed in the United States.

Impressed with this fact, the American Medical Association, a few years ago, unanimously adopted a resolution in which the establishment of such Schools was strongly recommended. In the Southern States, two have already been projected, and there can be little doubt that before many years they will be found wherever Medicine is systematically taught.

Acting upon the recommendation of the American Medical Association, the undersigned have united for the purpose of establishing an Institution of the kind,—a school, of which the object will be to impress upon the student's mind the elements of medical education, in conjunction with a practical knowledge of his profession.

The Marine Hospital, and the Hospital attached to the Alms House, afford an opportunity of studying a variety of cases at the bedside; and the Physicians* attached to those institutions, will give Clinical Instruction twice a week, or more frequently; the Roper Hospital, with accomodation for beds, it is hoped will be completed in a short time; and the most distinguished Surgeons of the city have kindly promised to operate in the presence of the students on such cases as may conveniently be brought before the class. In addition to which, the students will have access to all cases among the patients of the Teachers in the school, as they can with propriety be allowed to visit.†

Before each lecture, all members of the class, who do not object, will be examined thoroughly on the preceding lecture in the same department; and during the session of the Medical College of the State of South Carolina, they will be examined gratuitously on the lectures delivered at that Institution.

Hygienic improvements, vigorously carried on during many years past, have, there is reason to hope, now rendered Charleston entirely exempt from even those *occasional* epidemics of fever which formerly deterred strangers from visiting her in summer; and her mortuary statistics prove her to possess a salubrity of climate enjoyed by very few cities in the world.

The expense of living here is moderate. Board and lodging may be had in respectable houses from $3.50 to $5 a week, and upwards, according to the character of the accommodation furnished.

* Drs. D. J. Cain and J. F. Prioleau.

† Such cases as can be brought to the Lecture room will there be exhibited and explained.

Drs. R. Sobby and J. F. Mitchell, who have each a large Obstetrical Practice, will, as well as the regular teachers in the school, enable the student to see midwifery cases as frequently as possible. In short, ample opportunity will be afforded for acquiring a practical as well as theoretical knowledge of the profession.

A line of steamboats, making frequent and regular trips, enables one conveniently to attend to business in the city through the day, and to spend the evening and night in the enjoyment of the delightful atmosphere of Sullivan's Island.

The session of the School will commence on the first Monday in April, and terminate on the last Saturday in July.

The different Chairs will be occupied as follows:

*Anatomy and Physiology,*—By Dr. F. T. Miles.

*Institutes and Practice of Medicine,*—By Dr. D. J. Cain.

*Materia Medica and Therapeutics,*—By Dr. F. Peyre Porcher.

*Obstetrics and Diseases of Women and Children,*—By Dr. E. Belin Flagg.

The Chair of Surgery has been offered to a gentleman now in Europe. Should he accept it, his name will appear in another Prospectus a few weeks hence; should he not do so, the Chair will be otherwise filled as soon as an answer from him is received.

*Terms.*—Fifty dollars for the Course, including Examinations during the Winter.

N. B.—Further information can be obtained from Dr. Cain, No. 98 Wentworth Street, Dr. Flagg, No. 15 Archdale Street, Dr. Porcher, No. 30 Wentworth Street, or Dr. Miles, Beaufeiul Street.

